# Exposure to Herbicides Prime P450-Mediated Detoxification of *Helicoverpa armigera* against Insecticide and Fungal Toxin

**DOI:** 10.3390/insects10010028

**Published:** 2019-01-11

**Authors:** Zhongxiang Sun, Cuicui Xu, Shi Chen, Qi Shi, Huanhuan Wang, Rumeng Wang, Yuanyuan Song, Rensen Zeng

**Affiliations:** 1 College of Crop Science, Fujian Agriculture and Forestry University, Fuzhou 350002, China; szx@fafu.edu.cn (Z.S.); 18825197414@163.com (R.W.); 2 State Key Laboratory of Ecological Pest Control for Fujian and Taiwan Crops, Fuzhou 350002, China; 3 College of Life Science, Fujian Agriculture and Forestry University, Fuzhou 350002, China; 18452636956@163.com (C.X.); 15225935142@163.com (Q.S.); 15705989227@163.com (H.W.); 4 State Key Laboratory of Conservation and Utilization of Subtropical Agro-Bioresources, College of Agriculture, South China Agricultural University, Guangzhou 510642, China; goodnessstc@163.com

**Keywords:** herbicides, insecticides, antagonistic interaction, P450, *Helicoverpa armiger*

## Abstract

With the long-term and large-scale use, herbicides have been well known to influence tritrophic interactions, particularly natural enemies of pests in agro-ecosystems. On the other hand, herbivorous insects, especially the generalist pests, have developed antagonistic interaction to different insecticides, toxic plant secondary metabolites, and even heavy metals. However, whether exposure to herbicides would affect resistance of insects against insecticides is largely unknown, especially in agricultural pests. Here, we first reported that pre-exposure to two widely used herbicides butachlor and haloxyfop-methyl for 48 h can prime the resistance of a generalist agricultural pest *Helicoverpa armigera* Hübner against insecticide methomyl and fungal toxin aflatoxin B1. In addition, there were no significant differences between control and herbicides-treated caterpillars on weight gain, pupal weight, and pupation rates, suggesting that exposure to herbicides induces resistance of *H. armigera* accompanied with no fitness cost. Moreover, by determining detoxifying enzyme activities and toxicity bioassay with additional inhibitor of cytochrome P450 piperonyl butoxide (PBO), we showed that exposure to herbicides might prime P450-mediated detoxification of *H. armigera* against insecticide. Based on these results, we propose that exposure to herbicides prime resistance of *H. armigera* against insecticide and fungal toxin by eliciting a clear elevation of predominantly P450 monooxygenase activities in the midgut and fat body.

## 1. Introduction

The application of insecticides is currently the most common control measure against insect pests [1]. However, the resistance of insects to chemical insecticides has become a growing agricultural and ecological concern [2]. Since pesticides are widely and cross used in agriculture, one insecticide is known to confer resistance to other insecticides in insects through the cross-resistance mechanism [3,4].

Herbicides have been largely used in agriculture to control weeds around the world. However, excessive and inappropriate use of herbicides have also lead to serious harmful effects, including breaking ecological chain, increasing environmental pollution, and sanitation concerns [5,6]. For example, the herbicide glyphosate has great infection on the population numbers of non-target *Lepthyphantes Tenuis* [5,6]. Exposure to the herbicide atrazine at low ecologically relevant doses cause *Xenopus Laevis* hermaphroditic and demasculinized during sexual development [7]. What is worse, farmers and entomologists have observed that insects have evolved insecticide resistance when exposed to particular insecticides, including herbicides [8,9]. In mosquitoes, larvae that are exposed to the herbicide atrazine and benzothiazole become more tolerant to insecticides [4,10]. However, the antagonistic interaction between herbicides and insecticides is largely unknown in agricultural pests.

The induction of enzymatic activities, such as cytochrome P450 monooxygenases (P450), glutathion-S-transferase (GST), and esterases, are strongly associated with insecticide resistance in insects [11,12]. The detoxification enzyme system also take the major responsibility for cross-resistance mechanism [9,13]. For instance, the expression of multiple P450 and GST genes, which were previously linked to insecticide resistance, have been shown to be simultaneously induced by the herbicide atrazine [14].

*Helicoverpa armigera* Hübner (Lepidoptera: Noctuidae) is an economically important agricultural pest that is responsible for severe yield loss in more than 200 host plants [15]. The control of the *H. armigera* has been achieved with insecticides, such as methomyl and transgenic *Bt* crops. Methomyl is one kind of carbamate pesticides, which is structurally similar to the good inhibitors of acetylcholin esterase (AChE) and acetylcholine (ACH), cause excessive accumulation of ACH, and lead to interference with the normal conduction of nerve impulses in insects [16]. However, *H. armigera* has developed resistance to multiple classes of insecticides worldwide [17,18]. Aflatoxin B1 (AFB1) is a fungal metabolite that is produced by *Aspergillus flavus* and related fungi [19]. AFB1 requires metabolic activation by cytochrome P450 and then damage DNA, leading to strong carcinogenic mutagenesis acute toxicity in animals, including insects [19]. Butachlor (BuCh) and haloxyfop-methyl (HLFM) are both intensively used herbicides in a variety of plant crops, including rice, cotton, wheat peanuts, and cabbage crops [20,21]. Insects could be exposed to residual herbicides and AFB1 by feeding on host plants, and they are also subjected to pesticide stress in the ecosystem of cropland. However, little is known about the antagonistic interaction between herbicides that are used for plant crops and insecticides or fungal toxin used for *H. armigera*.

The aim of this study was to demonstrate that pre-exposure to herbicides BuCh or HLFM can prime the resistance of *H. armigera* against insecticide methomyl and fungal toxin aflatoxin B1 (AFB1). Furthermore, to identify which detoxification enzyme system took the major responsibility for herbicides induced resistance in *H. armigera*, the most universal detoxification enzymes, including cytochrome P450 monooxygenases, glutathion-S-transferase, and esterases in *H. armigera* were investigated to illuminate the possible antagonistic interaction mechanism between herbicides and insecticides.

## 2. Material and Methods

### 2.1. Insects

The laboratory strain of cotton bollworm (*Helicoverpa armigera*), provided by the Insectarium of the Institute of Entomology, Sun Yat-sen University, were reared on artificial diets that are composed of soybean powder (50 g), corn flour (40 g), brewer’s yeast (40 g), wheat bran (40 g), ascorbic acid (4 g), methyl p-hydroxybenzoate (1.8 g), sorbic acid (2 g), agar (20 g), casein (25 g), saccharose (20 g), streptomycin (0.15 g), 10% methanol (2 mL), and water (1 L), without exposure to any insecticide and were used for all experiments. The caterpillars were maintained at 25 ± 2 °C with 70 ± 5% relative humidity and a photoperiod of 14:10 h (L:D) in a climatic chamber. Adults were provided with 10% honey solution under the same conditions.

### 2.2. Chemicals

Methomyl 98% was purchased from Jiangsu Jinghong Chemical Co. Ltd (Yancheng, China); butachlor (BuCh) was purchased from Shandong qiaochang chemical Co. Ltd (Binzhou, China); haloxyfop-methyl (HLFM) was purchased from Dow AgroSciences LLC (Indianapolis, IN, USA); cytochrome P450 inhibitor piperonyl butoxide (PBO), aflatoxin B1 (AFB1) and dimethyl sulfoxide (DMSO) were purchased from Sigma (St. Louis, MO, USA); and, EDTA and Sodium dodecyl sulfate (SDS) were bought from Shanghai Health and Biotechnology Co., Ltd (Shanghai, China). Phenylmethylsulfonyl chloride (PMSF) was obtained from Beijing Dingguo Biotechnology Co., Ltd (Beijiing, China). Bovine serum albumin was bought from Shanghai Boao Biotechnology Co., Ltd (Shanghai, China). Dimercaptosyl alcohol (DDT) was purchased from Shanghai source poly biotechnology Co., Ltd (Shanghai, China).

### 2.3. Pre-Exposure to Herbicides and Bioassays

Since insects could be exposed to residual herbicides by feeding on host plants or by olfactory perception from their surroundings, we treated caterpillars with either volatile herbicides or diet containing herbicides to simulate the natural field conditions. Newly molted fifth-instar caterpillars of *H. armigera* were fed on a control diet (containing no allelochemicals) exposed to 1 μg volatile herbicides butachlor (BuCh) or haloxyfop-methyl (HLFM), or fed on diet containing 0.5 mg/g herbicides BuCh or HLFM exposing to fresh air for 48 h, and then accumulative mortalities were recorded 60, 120, 180, 240, 300, 360, and 720 min after treated with methomyl (50 μg per caterpillar) and the final mortalities were recorded at 1440 min. The control caterpillars were fed on control diet exposed to fresh air in the volatile induction experiment, and fed on diet containing 0.5 mg/g solvent sterilized water in the feeding experiment. In the synergism analysis, 3 μL cytochrome P450 inhibitor piperonyl butoxide (PBO) was delivered on to the prothorax notum of each caterpillar 1 h before the application of insecticide. Twenty synchronous fifth-instar caterpillars were used for each treatment, and three independent replicates were performed. For toxicity bioassay of carcinogenic mycotoxin aflatoxin B1 (AFB1), third-instar caterpillars were pre-exposed to BuCh or HLFM for 48 h, as mentioned above, and the caterpillars were fed on diet containing 2.5 μg/g AFB1. After that, the weight gain from the third-instar to sixth-instar caterpillars and final mortalities were recorded. Twenty synchronous third-instar caterpillars were used for each treatment, and three independent replicates were performed.

To investigate the effects of exposing to herbicides on growth and development of *H. armigera*, third-instar caterpillars were used, and the weight gain from the third-instar to sixth-instar caterpillars, pupal weight and pupation rates were compared between the control group and herbicides exposing group. Twenty synchronous third-instar caterpillars were used for each treatment, and three independent replicates were performed.

### 2.4. Enzyme Activity Assay

To assay the detoxification enzyme activities, the total midgut and fat body from BuCh- or HLFM-treated caterpillars of *H. armigera* were used. Newly molted fifth-instar caterpillars were fed on a control diet (containing no allelochemicals) exposed to 1 μg volatile herbicides butachlor (BuCh) or haloxyfop-methyl (HLFM), or fed on diet containing 0.5 mg/g herbicides BuCh or HLFM exposing to fresh air for 48 h and then the midgut and fat body were dissected on an ice plate and placed in 0.1 mM phosphate buffer solution (PBS, pH 7.0, containing 1 mmol/L EDTA, 0.1 mmol/L PMSF, 0.1 mmol/L DTT and 10% glycerol). The homogenate was centrifuged at 4 °C, 10,000 g for 20 min. The supernatant of each treatment was used immediately for enzyme assays or stored at −80 °C until use. The determination of cytochrome P450 enzyme activities were performed according to previously published procedures with slight modifications [22]. The determination of the glutathione S-transferase was slightly modified by the measure of Habig et al [23]. The determination of the carboxylesterase was slightly modified by the measure of Van Asperen [24]. For the acetylcholinesterase activity, the midgut or fat body was ground in 2 mL of 0.70% NaCl and centrifuged at 4 °C, 3500 g for 10 min. The supernatant was used for enzyme assays by using a Choline esterase (ChE) detection kit (Nanjing Jiancheng Bioengineering). Twenty synchronous fifth-instar caterpillars were used for each treatment, and three independent replicates were performed for all treatments.

### 2.5. Statistical Analysis

All data are presented as mean ± standard error. For statistical evaluation of the experiments, one-way analysis of variance was performed. Different letters indicate significant differences (*p* < 0.05) according to Tukey’s multiple range test. All of the data analyses were performed using GraphPad Prism v.6.01 (GraphPad software, Inc., San Diego, CA, USA).

## 3. Results

### 3.1. Exposure to Herbicides Prime Resistance of H. armigera against Insecticide

**Figure 1:**
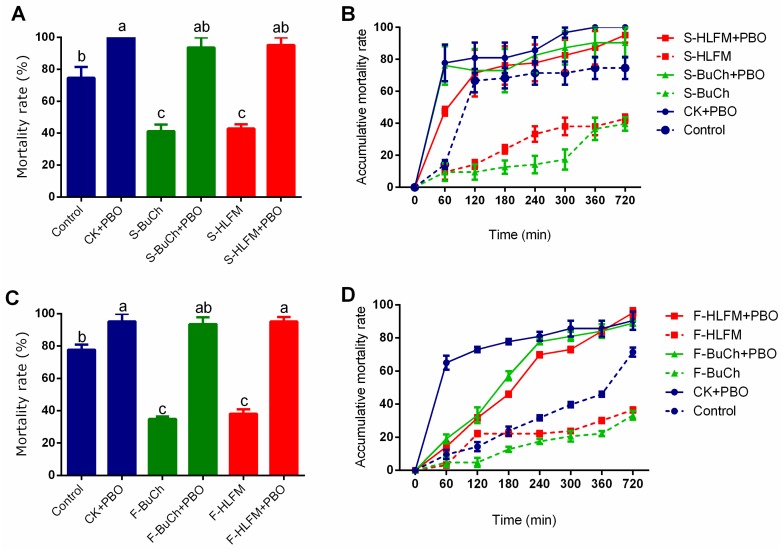
Effects of volatile smelling and diet feeding of herbicides on mortality of *H. armigera* caterpillars treated with methomyl. Mortality rates (**A**) and Accumulative mortality rates (**B**) of *H. armigera* caterpillars treated with methomyl after pre-exposed of volatile herbicides. Mortality rates (**C**) and Accumulative mortality rates (**D**) of *H. armigera* caterpillars treated with methomyl after diet feeding of herbicides. Newly molted fifth-instar caterpillars were fed on control diet (containing no allelochemicals) exposing to volatile herbicides or fed on diet containing herbicides exposing to fresh air for 48 h, then caterpillars were treated with methomyl (50 μg per caterpillar) and the mortalities were recorded on 60, 120, 180, 240, 300, 360, 720, and 1440 min. S-BuCh, caterpillars fed on control diet exposing to 1 μg volatile butachlor; S-HLFM, caterpillars fed on control diet exposing to 1 μg volatile haloxyfop-methyl; F-BuCh, caterpillars fed on diet containing 0.5 mg/g butachlor; F-HLFM, caterpillars fed on diet containing 0.5 mg/g haloxyfop-methyl; “+ PBO” represent that caterpillars were pre-treated with cytochrome P450 inhibitor piperonyl butoxide (PBO 3 μL per caterpillar) 1 h earlier before methomyl treatment. Data shown are mean ± SE (n = 3). Twenty synchronous fifth-instar caterpillars were used for each treatment, and three independent replicates were performed. Different letters indicate significant differences (*p* < 0.05), according to Tukey’s multiple range test.

We performed toxicity bioassays to evaluate the effects of pre-exposure to butachlor (BuCh) or haloxyfop-methyl (HLFM) on susceptibility of *H. armigera* caterpillars to methomyl. Since insects could be exposed to residual herbicides by feeding on host plants or by olfactory perception from their surroundings, we treated caterpillars with either volatile herbicides or diet containing herbicides to simulate the natural field conditions. We found that induction of *H. armigera* caterpillars with either BuCh or HLFM, and whatever kinds of treatment, significantly decreased caterpillars mortality to methomyl, from ~80% (Control) to ~40% (S-BuCh, S-HLFM, F-BuCh, and F-HLFM) (Figure 1A,C, *p* < 0.05). Moreover, kinetics of *H. armigera* mortality follow the same pattern (Figure 1B,D). These results showed that exposure to herbicides prime resistance of *H. armigera* against insecticide.

Since piperonyl butoxide (PBO) is an inhibitor of cytochrome P450 and is used as a representative synergist, we tested the synergistic effects of herbicides and PBO on methomyl toxicities. Remarkably, larvae pre-treated with PBO before the insecticide application (BuCh + PBO, HLFM + PBO) significantly increased both the mortality rates and accumulative mortality rates in all treatment groups (Figure 1), which imply that induced resistance of *H. armigera* against insecticide was counteracted by PBO treatment. These encouraging results showed that exposure to herbicides might prime P450-mediated detoxification of *H. armigera* against insecticide.

### 3.2. Exposure to Herbicides Prime Resistance of H. armigera against Fungal Toxin

**Figure 2:**
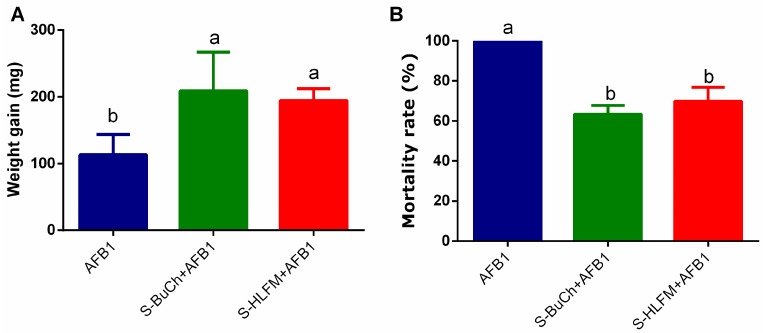
Effects of volatile smelling of herbicides on AFB1 resistance of *H. armigera*. Weight gain (**A**) and Mortality rates (**B**) of *H. armigera* caterpillars reared on Aflatoxin B1 (AFB1)-supplemented diet after pre-exposed of volatile herbicides. Newly molted third-instar caterpillars were fed on control diet (containing no allelochemicals) exposing to volatile herbicides for 48 h, and weight gain from the third-instar to sixth-instar caterpillars and final mortalities were recorded were recorded after exposing to 2.5 μg/g AFB1. S-BuCh, caterpillars exposing to 1 μg volatile butachlor; S-HLFM, caterpillars exposing to 1 μg volatile haloxyfop-methyl. Data shown are mean ± SE (n = 3). Twenty synchronous individuals were used for each treatment and three independent replicates were performed. Different letters indicate significant differences (*p* < 0.05) according to Tukey’s multiple range test.

We also performed toxicity bioassay to evaluate the effects of pre-exposed to BuCh or HLFM on the susceptibility of *H. armigera* caterpillars to carcinogenic mycotoxin aflatoxin B1 (AFB1). The weight gain of either BuCh or HLFM exposed caterpillars were significantly higher than control caterpillars (Figure 2A, *p* < 0.05), and the mortality rates of either BuCh or HLFM exposed caterpillars were significantly lower than the control (Figure 2B, *p* < 0.05). These results imply that exposure to herbicides prime resistance of *H. armigera* against AFB1.

### 3.3. The Effects of Exposing to Herbicides on Growth and Development of H. armigera

**Figure 3:**
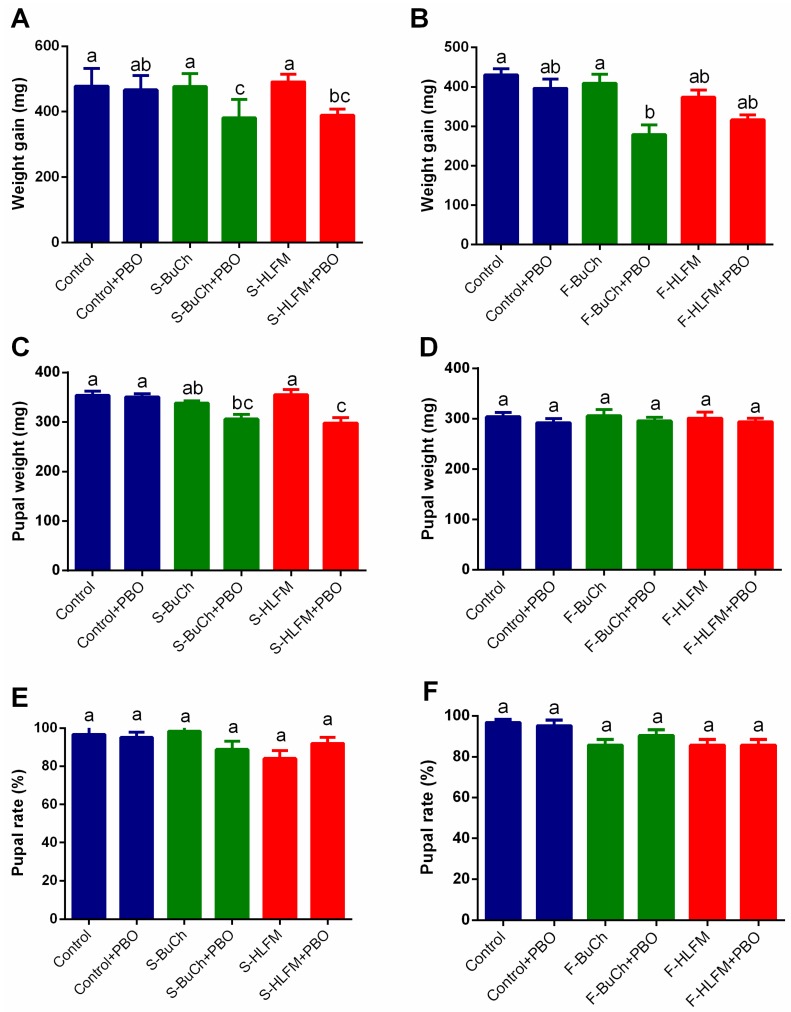
Effects of volatile smelling and diet feeding of herbicides on caterpillar growth and development of *H. armigera*. Weight gain (**A**) Pupal weight (**C**) and Pupal rate (**E**) of *H. armigera* caterpillars reared on control diet (containing no allelochemicals) after pre-exposed of volatile herbicides. Weight gain (**B**) Pupal weight (**D**), and Pupal rate (**F**) of *H. armigera* caterpillars reared on diet containing herbicides. Newly molted third-instar caterpillars were fed on control diet (containing no allelochemicals) exposed to volatile herbicides or fed on diet containing herbicides for 48 h, and weight gain, pupal weight, and pupal rate were recorded. S-BuCh, caterpillars fed on control diet exposing to 1 μg volatile butachlor; S-HLFM, caterpillars fed on control diet exposing to 1 μg volatile haloxyfop-methyl; F-BuCh, caterpillars fed on diet containing 0.5 mg/g butachlor; F-HLFM, caterpillars fed on diet containing 0.5 mg/g haloxyfop-methyl; “+ PBO” represent that caterpillars were pre-treated with cytochrome P450 inhibitor piperonyl butoxide (PBO 3 μL per caterpillar) 1 h earlier before methomyl treatment. Twenty synchronous individuals were used for each treatment and three independent replicates were performed. Data shown are mean ± SE (n = 3). Different letters indicate significant differences (*p* < 0.05), according to Tukey’s multiple range test.

To investigate the effects of exposing to herbicides on growth and development of *H. armigera*, the weight gain of third-instar caterpillars, pupal weight, and pupation rates were compared between control group and herbicides exposing group. As shown in Figure 3, there were no significant differences between control group (Control) and herbicides treatments (S-BuCh, S-HLFM, F-BuCh, and F-HLFM) on weight gain, pupal weight, and pupation rates (Figure 3, *p* > 0.05). Although three treatments pre-treated with PBO showed slightly lower than the larvae without PBO pre-treating (Figure 3A–C, S-BuCh + PBO vs. S-BuCh, S- HLFM + PBO vs. S- HLFM and F-BuCh + PBO vs. F-BuCh on weight gain; S- HLFM + PBO vs. S- HLFM on pupal weight), most treatments showed no significant differences. These results suggest that exposure to herbicides did not affect growth and development of *H. armigera* and induce resistance of *H. armigera* accompanied with no fitness cost.

### 3.4. The Effect of Exposing to Herbicides on Detoxifying Enzyme Activities in H. armigera

To identify which detoxification enzyme system took the major responsibility for herbicides induced resistance in *H. armigera*, four kinds of universal detoxification enzymes, including cytochrome P450 monooxygenases (P450), glutathione S-transferase towards 3,4-dichloronitrobenzene (GST-DCNB), esterase activity towards a-naphthyl acetate (Esterase-aNA), and choline esterase (ChE) in both the midgut and fat body of *H. armigera* were investigated. In the assay of P450 in the midgut, the activities in the herbicides-treated group were extremely increased by 1.7-fold to 4.8-fold (Table 1). For the GST-DCNB, the activities were slightly inhibited in the BuCH-treated group, but increased in the S-HLFM-treated group (Table 1). For Esterase-aNA, the activities were slightly inhibited in the BuCH-treated group and more significantly inhibited in the F-HLFM-treated group (Table 1). For ChE, no obvious differences of midgut activities were observed between the herbicides -treated and the control caterpillars (Table 1).

For the activities of detoxification enzymes in fat body of *H. armigera*, similar results were obtained for P450, the activities in the herbicides-treated group were extremely increased by 2.1-fold to 2.6-fold (Table 2). For the GST-DCNB, the activities were slightly inhibited in the BuCH-treated group, but increased in the F-HLFM-treated group (Table 2). For Esterase-aNA, the activities were significantly increased in all herbicides-treated groups (Table 2). For ChE, the activities were inhibited in the S-BuCH-treated group and the F-HLFM-treated group (Table 2).

Taken together, these data demonstrate that exposure to herbicides elicited a clear elevation of predominantly P450 monooxygenase activities in the midgut and fat body.

## 4. Discussion

Most of the important agricultural pests are polyphagous, which can feed on a variety of host plants [25] and develop resistance to a variety of pesticides [26,27]. For example, the cotton bollworms (*Helicoverpa armigera*) is a major polyphagous pest feeding on hundreds of different plants, including cotton (Gossypium hirsutum), and show rapid evolution of resistance against various kinds of insecticides, even the transgenic *Bacillus thuringiensis* (Bt) cotton [28,29]. Therefore, to research how the polyphagous insect copes with the diversity of plant defenses and pesticides is of great concern in crop protection.

Cross-resistance is the tolerance to a usually toxic substance as a result of exposure to a similarly acting substance, which is quite ubiquitous in insects [30]. Especially, when adapting to a wide variety of host plants and adventurous chemical environment, many herbivorous generalist insects have developed antagonistic interaction to insecticides and toxic plant secondary metabolites [31,32]. *H. armigera* can take advantage of gossypol from cotton plants to elaborate defense systems against a pyrethroid insecticide [31]. The most significant case is the cross-resistance between imidacloprid, thiamethoxam, and acetamiprid in the *Bemisia tabaci* [32]. With the long-term and large-scale use, herbicides have already been well known to influence wild plant diversity in agro-ecosystems, tritrophic interactions, particularly natural enemies of pests and environmental contamination [33]. However, whether exposure to herbicides would affect insecticides resistance is largely unknown, especially in agricultural pests. In this study, we showed that exposure to herbicides butachlor (BuCh) or haloxyfop-methyl (HLFM) can prime the resistance of *H. armigera* against insecticide and fungal toxin. Although mosquitoes such as *Aedes aegypti* has been found increasing tolerance to insecticide temephos after exposure to atrazine [34], to our knowledge, there is scarcely any report that antagonistic interaction between herbicides and insecticides is also exist in the agricultural pest. We also found that being pre-exposed to BuCh or HLFM significantly decreases the susceptibility of *H. armigera* caterpillars to carcinogenic mycotoxin aflatoxin B1 (AFB1). These results suggest that *H. armigera* can take advantage of pre-exposed to herbicides for antagonistic interaction with wide targets. We speculated that it is a very significant reason for *H. armigera* being a polyphagous pest on many plants and in complex environmental stresses.

The development of resistance to an insecticide in insects are generally associated with high energetic cost or significant disadvantage when compared with susceptible individuals [35]. For example, fitness costs of Bt resistance occur in many species of insects, that is, in the absence of Bt toxins, fitness is lower for resistant insects than for susceptible insects [36]. In this study, our preliminary findings suggest that exposure to herbicides did not affect growth and development of *H. armigera* and induce resistance of *H. armigera* against insecticide and fungal toxin aflatoxin B1 accompanied with low fitness cost. More fitness effects, such as adult emergence rate, adult fecundity, and longevity, should be studied in the future. A large number of studies have shown that multiple, complex resistance mechanisms are responsible for insecticide resistance in insects [37], including increased metabolic detoxification of insecticides, decreased sensitivity of the target proteins, and reduction of cuticular penetration [38,39,40,41]. Insect detoxification enzymes typically include three main superfamilies: cytochrome P450 monooxygenases (P450s), glutathione S-transferases (GSTs), and carboxylesterases (CarEs) [12]. Since the detoxification enzyme system takes the major responsibility for cross-resistance mechanism [9,13]. We successively measure the major detoxifying enzyme activities after exposure to herbicides on in both the midgut and fat body of *H. armigera*. Interestingly, exposure to herbicides, either BuCh or HLFM, elicited a clear elevation of predominantly P450 monooxygenase activities in both midgut and fat body. The up-regulation of P450 genes mediated by insecticides and other xenobiotic compounds have been largely reported in insect species [42,43]. Multiple P450 genes have been reported to be overexpressed in insecticides-resistant strains in *H. armigera*, especially the CYP4 clan, CYP6, and CYP9 families [18,44,45]. Therefore, the changes in transcript abundance of P450 genes after exposure to herbicides should be systematically studied in a follow-up experiment.

In order to develop an effective long-term resistance management strategy, it is very necessary to monitor the resistance and cross-resistance between different insecticides [30]. When considering that insects are exposed to chemical insecticides and meanwhile could be exposed to residual herbicides by feeding on host plants or by olfactory perception from their surroundings, what we find here could be one potential risk of priming pest insecticides resistance by antagonistic interaction with herbicides. Therefore, we argue that detailed study of the impacts of herbicides on insect will facilitate efforts to reduce the influence of antagonistic interaction in pest control in the future.

## 5. Conclusions

In conclusion, we propose that exposure to herbicides prime resistance of *H. armigera* against insecticide by eliciting a clear elevation of predominantly P450 monooxygenase activities in the midgut and fat body.

## Figures and Tables

**Table 1:** Activities of detoxification enzymes in midgut of *H. armigera*.

	P450	GST-DCNB	Esterase-aNA	ChE
	(nmole per min per mg pro)			
CK	0.134 ± 0.009 d	4.22 ± 0.06 b	37.53 ± 1.33 a	16.07 ± 2.76 a
S-BuCH	0.230 ± 0.016 c	3.19 ± 0.13 c	30.03 ± 0.63 b	20.14 ± 2.28 a
F-BuCH	0.327 ± 0.020 bc	3.38 ± 0.12 c	33.03 ± 0.45 b	19.97 ±1.23 a
S-HLFM	0.383 ± 0.015 b	5.10 ± 0.11 a	40.98 ± 0.58 a	22.04 ± 1.85 a
F-HLFM	0.650 ± 0.034 a	4.87 ± 0.23 ab	17.14 ± 0.57 c	21.48 ± 1.50 a

CK, caterpillars fed on control diet without exposing to herbicides; S-BuCh, caterpillars fed on control diet exposing to 1 μg volatile butachlor; S-HLFM, caterpillars fed on control diet exposing to 1 μg volatile haloxyfop-methyl; F-BuCh, caterpillars fed on diet containing 0.5 mg/g butachlor; F-HLFM, caterpillars fed on diet containing 0.5 mg/g haloxyfop-methyl. Twenty synchronous individuals were used for each treatment, and three independent replicates were performed. Data shown are mean ± SE (n = 3). Different letters indicate significant differences (*p* < 0.05) according to Tukey’s multiple range test.

**Table 2:** Activities of detoxification enzymes in fat body of *H. armigera*.

	P450	GST-DCNB	Esterase-aNA	ChE
	(nmole per min per mg pro)			
CK	0.130 ± 0.034 b	3.37 ± 0.33 b	7.18 ± 0.35 c	16.06 ± 0.81 a
S-BuCH	0.270 ± 0.027 a	2.66 ± 0.27 c	11.87 ± 0.57 b	8.19 ± 0.84 b
F-BuCH	0.317 ± 0.018 a	2.20 ± 0.18 c	14.16 ± 0.43 b	17.02 ± 1.01 a
S-HLFM	0.343 ± 0.028 a	3.69 ± 0.33 b	22.24 ± 0.27 a	13.79 ± 1.64 a
F-HLFM	0.284 ± 0.054 a	4.44 ± 0.13 a	14.78 ± 1.37 b	7.24 ± 0.23 b

CK, caterpillars fed on control diet without exposing to herbicides; S-BuCh, caterpillars fed on control diet exposing to 1 μg volatile butachlor; S-HLFM, caterpillars fed on control diet exposing to 1 μg volatile haloxyfop-methyl; F-BuCh, caterpillars fed on diet containing 0.5 mg/g butachlor; F-HLFM, caterpillars fed on diet containing 0.5 mg/g haloxyfop-methyl. Twenty synchronous individuals were used for each treatment, and three independent replicates were performed. Data shown are mean ± SE (n = 3). Different letters indicate significant differences (*p* < 0.05) according to Tukey’s multiple range test.
